# Adrenal Insufficiency‐Induced Delirium Following Gastrectomy in a Patient With Gastric Cancer Treated With Nivolumab, Immune Checkpoint Inhibitors: A Case Report

**DOI:** 10.1002/npr2.70072

**Published:** 2025-11-11

**Authors:** Kazuki Yamada, Taro Sasaki, Tamaki Owada, Kota Kikuchi, Ryo Maehara, Yasushi Kawamata, Norio Sugawara, Norio Yasui‐Furukori

**Affiliations:** ^1^ Department of Psychiatry Dokkyo Medical University School of Medicine Tochigi Japan

**Keywords:** adrenal insufficiency, delirium, gastrectomy, immune checkpoint inhibitors, immune‐related adverse events, nivolumab, psychoneuroendocrinology

## Abstract

**Background:**

Delirium is common after major surgery, yet endocrine causes such as adrenal insufficiency (AI) may be underrecognized, particularly in patients previously exposed to immune checkpoint inhibitors (ICIs); recent guidance encourages systematic hormonal monitoring (e.g., morning cortisol/ACTH) during ICI therapy.

**Case Presentation:**

We present the case of a 69‐year‐old female who developed hyperactive delirium following total gastrectomy for previously treated gastric cancer with nivolumab. Persistent hypotension and hypoglycemia prompted endocrine testing, which revealed low morning cortisol with inappropriately low ACTH, consistent with secondary AI. Brain MRI and EEG showed no alternative etiologies. Dynamic testing could not be performed in the acute setting, and a dedicated preoperative HPA‐axis screen had not been undertaken. Hydrocortisone replacement therapy resulted in rapid resolution of neuropsychiatric and systemic symptoms.

**Conclusion:**

This case highlights adrenal insufficiency as an underrecognized cause of delirium in ICI‐treated patients during the perioperative period. Awareness, early endocrine evaluation, and timely glucocorticoid replacement are crucial; preoperative screening may be considered in ICI‐exposed patients scheduled for major surgery.

## Abstract 2

Postoperative hyperactive delirium unmasked secondary adrenal insufficiency in a patient with prior PD‐1 inhibitor exposure. Surgical stress revealed ICI‐related hypophysitis (↓ACTH → ↓cortisol). Hypotension and hypoglycemia prompted endocrine testing (AM cortisol 1.01 μg/dL; ACTH 1.5 pg/mL). Hydrocortisone replacement led to rapid resolution and antipsychotic de‐escalation. Consider preoperative HPA‐axis screening in ICI‐exposed surgical candidates.

## Graphical abstract

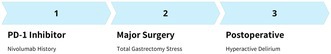

## 1 Introduction

Delirium, an acute neuropsychiatric syndrome involving disturbances in attention, awareness, and cognition, is frequently encountered in hospitalized patients, especially following major surgery [1]. While its etiologies are multifactorial, endocrine disturbances, such as adrenal insufficiency, are increasingly recognized as important yet often overlooked contributors [2].

The advent of immune checkpoint inhibitors (ICIs), particularly PD‐1 inhibitors such as nivolumab, has dramatically improved outcomes for patients with advanced gastric cancer [3]. However, ICIs can induce a range of immune‐related adverse events (irAEs) affecting multiple organ systems [4]. Endocrine irAEs, including hypophysitis and secondary adrenal insufficiency, are less common with PD‐1 inhibitors than with cytotoxic T‐lymphocyte‐associated protein 4 (CTLA‐4) inhibitors but remain clinically significant [5, 6, 7, 8].

Adrenal insufficiency may remain subclinical until triggered by physiological stress, such as surgery [9, 10]. Neuropsychiatric manifestations of adrenal insufficiency are diverse and can include mood disorders, cognitive dysfunction, psychosis, and delirium [11, 12]. Delirium caused by adrenal insufficiency, particularly in the context of ICIs, has rarely been reported [13, 14, 15, 16, 17].

Endocrine immune‐related adverse events from ICIs include hypophysitis, which can lead to secondary adrenal insufficiency. These events are less frequent with PD‐1 inhibitors than with CTLA‐4 blockade but can be subtle or delayed, even after discontinuation of ICIs, and can be triggered by physiological stressors such as major surgery. According to contemporary guidance, periodically monitoring morning cortisol and ACTH levels (as well as thyroid function) during ICI therapy may help detect subclinical adrenal insufficiency before operative stress [18, 19, 20].

We present a unique case of adrenal insufficiency–induced hyperactive delirium following gastrectomy in a patient who had previously been treated with nivolumab for gastric cancer. This case highlights the diagnostic challenges and therapeutic implications.

## 2 Case Presentation

A 69‐year‐old woman with advanced gastric cancer underwent 4 cycles of capecitabine, oxaliplatin, and nivolumab (CapeOX) after a biopsy confirmed peritoneal dissemination. After treatment, the peritoneal lesions resolved, and a curative total gastrectomy with cholecystectomy was performed. Following surgery, however, she developed mutism, anorexia, generalized weakness, an unsteady gait, and progressive behavioral changes. A psychiatric evaluation upon readmission revealed disorganized speech, flight of ideas, hyperactivity, emotional lability, and psychomotor agitation. No hallucinations or delusions were observed. These clinical features met the DSM‐5 diagnostic criteria for delirium, characterized by acute onset, fluctuating course, disturbances in attention, awareness, and cognition, without evidence of other primary psychiatric disorders [21]. Initial laboratory tests showed: sodium 136 mmol/L, potassium 3.8 mmol/L, chloride 109 mmol/L, CRP 2.16 mg/dL, albumin 2.7 g/dL, total protein 5.5 g/dL, HbA1c 5.7%, and blood glucose 47 mg/dL (severe hypoglycemia). Brain MRI demonstrated only age‐appropriate cortical atrophy; EEG showed preserved posterior alpha rhythm without epileptiform discharges. No evidence of structural brain lesions, CNS infection, or metabolic encephalopathy was found, supporting the diagnosis of delirium. A provisional diagnosis of brief psychotic disorder or delirium was initially considered. Risperidone (4 mg/day) was started but was reduced to 3 mg/day due to sedation, without significant improvement. Persistent hypotension (systolic BP < 90 mmHg), hypoglycemia, and poor oral intake raised suspicion for adrenal insufficiency. Endocrinological evaluation revealed: cortisol 1.01 μg/dL (ref 5–25), ACTH 1.5 pg/mL (ref 7.2–63.3), free T3 4.21 pg/mL, free T4 1.0 ng/dL, TSH 1.163 μIU/mL. These findings confirmed secondary adrenal insufficiency, likely due to nivolumab‐induced hypophysitis. A dedicated preoperative endocrine screening of the hypothalamic–pituitary–adrenal axis was not performed. Dynamic testing, such as cosyntropin stimulation, could not be performed in the acute phase due to clinical instability. Hydrocortisone replacement therapy (IV followed by 15 mg/day orally) led to stabilization of blood pressure (> 110/70 mmHg), resolution of hypoglycemia, improved oral intake, and gradual improvement in neuropsychiatric symptoms. Follow‐up MRI and EEG remained normal. Risperidone was successfully tapered off without recurrence. The patient was discharged after 49 days with complete neuropsychiatric recovery and was referred for continued outpatient follow‐up with endocrinology and psychiatry.

## 3 Discussion

In the present case, the diagnosis of delirium was established based on standard diagnostic criteria, including disturbance in attention, awareness, and cognition, with an acute onset and fluctuating course, in accordance with the DSM‐5 diagnostic criteria for delirium [21]. No evidence of alternative causes such as structural central nervous system disease, central nervous system infection, or toxic–metabolic etiologies unrelated to endocrine failure was identified; while metabolic abnormalities (notably hypoglycemia) were present, they were attributable to adrenal insufficiency rather than an independent cause. Importantly, delirium occurred in the immediate postoperative period in a patient previously exposed to a PD‐1 inhibitor (nivolumab), and physiological stress likely unmasked subclinical endocrine dysfunction. ICIs, including anti‐PD‐1 and anti‐CTLA‐4 agents, are increasingly recognized to induce various neuropsychiatric immune‐related adverse events, including encephalitis, aseptic meningitis, and, less frequently, delirium [4, 5, 9].

### 3.1 Diagnostic Clarity and Differential Diagnosis of Postoperative Delirium

We systematically considered alternative precipitants, including metabolic/electrolyte disturbances, infection/inflammation, structural etiologies, epileptiform etiologies, and drug‐induced causes. Together, the patient's laboratory values, MRI, and EEG (preserved posterior alpha and no epileptiform discharges) supported delirium secondary to endocrine failure precipitated by surgical stress, as did the hemodynamic/biochemical profile (hypotension and neuroglycopenia).

### 3.2 Basis for ICI‐Related Hypophysitis and Central Adrenal Insufficiency

Low morning cortisol levels and low ACTH levels indicated secondary adrenal insufficiency (AI). Other pituitary axes were not suppressed, and imaging showed no alternative intracranial processes. The brisk, sustained response to physiologic hydrocortisone supports a diagnosis of probable ICI‐induced hypophysitis with isolated ACTH deficiency. We acknowledge the following limitations: dynamic testing was not feasible acutely, pituitary imaging lacked specific inflammatory changes, and a dedicated preoperative HPA axis screen was not undertaken. These features and recommendations align with contemporary reviews and practice guidelines on endocrine irAEs [9, 10, 18, 19, 20].

### 3.3 Comparison With Prior Literature and Clinical Implications

Reports describe delayed‐onset PD‐1‐related hypophysitis/secondary autoimmune (AI) disease and postoperative unmasking after prior PD‐1 exposure [15, 16, 17]. Delirium as the initial neuropsychiatric manifestation is sparsely documented; however, AI can present with acute confusional states that resolve with glucocorticoid replacement [11, 13, 14]. For patients undergoing major surgery who have been exposed to ICIs, we recommend considering preoperative HPA axis screening and an early endocrine consultation. This approach is consistent with current guidance on monitoring and managing endocrine irAEs [18, 19, 20] and can help reduce missed subclinical AI and perioperative decompensation.

## 4 Conclusion

When diagnosing delirium in cancer patients receiving ICIs, clinicians should consider adrenal insufficiency, especially if it is accompanied by systemic signs such as hypotension, anorexia, and hypoglycemia. An early endocrinological evaluation and prompt steroid replacement therapy can lead to a rapid recovery and may prevent the need for antipsychotic medications. As ICIs are used more widely across malignancies, it is crucial to be aware of delayed and atypical endocrine irAEs, including neuropsychiatric complications, to ensure multidisciplinary care [9, 10, 18, 19, 20].

## Author Contributions

K.Y., T.S. and T.O. were involved in the clinical investigations. K.Y. and N.Y.‐F. wrote the manuscript. K.Y., T.S., T.O., R.M., Y.K., S.N. and N.Y.‐F. were involved in the literature review. All the authors have read and approved the final manuscript.

## Ethics Statement

The ethics committee of the School of Medicine at Dokkyo Medical University determined that there was no need to review this case.

## Consent

Written informed consent was obtained from the patient's family for the publication of this case report.

## Conflicts of Interest

The authors declare that they have no competing interests to report. Norio Yasui‐Furukori is an editorial board member of Neuropsychopharmacology Reports and a coauthor of this article. To minimize bias, they were excluded from all editorial decision‐making related to the acceptance of this article for publication.

## Data Availability

Data sharing is not applicable to this article, as no datasets were generated or analyzed during the current study.
